# The impact of COVID‐19 on nurses (ICON) survey: Nurses' accounts of what would have helped to improve their working lives

**DOI:** 10.1111/jan.15442

**Published:** 2022-09-29

**Authors:** Jane Ball, Sydney Anstee, Keith Couper, Jill Maben, Holly Blake, Janet E. Anderson, Daniel Kelly, Ruth Harris, Anna Conolly, the full ICON Study Team

**Affiliations:** ^1^ University of Southampton Southampton UK; ^2^ NIHR ARC Wessex Southampton UK; ^3^ Warwick Medical School University of Warwick Coventry UK; ^4^ Critical Care Unit University Hospitals Birmingham NHS Foundation Trust Birmingham UK; ^5^ School of Health Sciences University of Surrey Guildford UK; ^6^ School of Health Sciences University of Nottingham Nottingham UK; ^7^ NIHR Nottingham Biomedical Research Centre Nottingham UK; ^8^ Monash University Melbourne Victoria Australia; ^9^ Cardiff University Cardiff UK; ^10^ King's College London London UK

**Keywords:** content analysis, COVID‐19, midwives, morale, nurses, nursing, personal protective equipment, survey, wellbeing, work‐lives

## Abstract

**Aims:**

To use nurses' descriptions of what would have improved their working lives during the first peak of the COVID‐19 pandemic in the UK.

**Design:**

Analysis of free‐text responses from a cross‐sectional survey of the UK nursing and midwifery workforce.

**Methods:**

Between 2 and 14 April 2020, 3299 nurses and midwives completed an online survey, as part of the ‘Impact of COVID‐19 on Nurses’ (ICON) study. 2205 (67%) gave answers to a question asking for the top three things that the government or their employer could do to improve their working lives. Each participants' response was coded using thematic and content analysis. Multiple response analysis quantified the frequency of different issues and themes and examined variation by employer.

**Results:**

Most (77%) were employed by the National Health Service (77%) and worked at staff or senior staff nurse levels (55%). 5938 codable responses were generated. Personal protective equipment/staff safety (60.0%), support to workforce (28.6%) and better communication (21.9%) were the most cited themes. Within ‘personal protective equipment’, responses focussed most on available supply. Only 2.8% stated that nothing further could be done. Patterns were similar in both NHS and non‐NHS settings.

**Conclusions:**

The analysis provided valuable insight into key changes required to improve the work lives of nurses during a pandemic. Urgent improvements in provision and quality of personal protective equipment were needed for the safety of both workforce and patients.

**Impact:**

Failure to meet nurses needs to be safe at work appears to have damaged morale in this vital workforce. We identified key strategies that, if implemented by the Government and employers, could have improved the working lives of the nursing and midwifery workforce during the early stages of the COVID‐19 pandemic and could prevent the pandemic from having a longer‐term negative impact on the retention of this vital workforce.

**Patient or Public Contribution:**

No Patient or Public Contribution, due to the COVID‐19 Pandemic, urgency of the work and the target population being health and social care staff.

## 1 INTRODUCTION/BACKGROUND

As we face a global shortage of registered nurses, employers and governments should have a strong motivation to attend to the growth and sustainability of the nursing workforce, to boost retention and ensure nursing staff can deliver high‐quality care across the health and social care sector effectively and efficiently (Buchan, Catton, & Shaffer, 2022; WHO, 2020). Creating effective nursing workforce retention policies (or policy bundles) relies on an understanding of the needs and motivations of the workforce under different conditions (Buchan, Shaffer, & Catton, 2018).

The COVID‐19 pandemic has put global health and care systems under extraordinary pressure, both exacerbating and highlighting pre‐existing staff shortages (Beech et al., 2019; WHO, 2020) and exposing long‐standing deficits and strains in the NHS workforce (Ball, 2020). The pace and ease of virus transmission, and potential rapid escalation of symptoms to severe respiratory illness requiring urgent access to intensive care, led to health systems to reconfigure services to avoid being overwhelmed. Experiences in Northern Italy in March 2020 sent shock waves around the globe; the fine line between a system ‘coping’ and ‘not coping’ was revealed to be perilously thin; especially in those systems operating with little spare capacity (Bosa et al., 2022). One country after another announced emergency measures and the shutdown of all but essential movement and services became known as ‘lock‐downs’. In the UK, the public message was clear: ‘Stay home to protect the NHS’ (Gov.UK, 2020b). The first lockdown in the UK began on 23 March 2020. By 12 April 2020, the number of hospitalized COVID‐19 patients in the UK had reached 57,650 and COVID‐19 was recorded on the death certificates of 19,861 people (Gov.UK, 2020a).

In the UK, legal sanctions, such as police fines, were imposed on those that did not comply with lockdown laws. In addition, public announcements and media campaigns were used to highlight the importance of public health measures, such as social distancing, to minimize virus spread and reduce the risk of health services being overwhelmed. Public reporting of the steep rise in the numbers of people reported daily to have died from COVID‐19 underscored the severity of the existing threat. Media coverage of ambulances queuing around the block, warehouse‐style Intensive Therapy Units (ITUs), and health workers under intense pressure, heightened public awareness of the risks of the rapidly accelerating public health crisis (BBC, 2021a, 2021b).

It was within this context of risk and alarm that nurses, midwives, carers and other health care professionals were expected, and were desperately needed, to continue their work, caring for increasing numbers of acutely ill‐patients with COVID‐19. Public gratitude for the work of health and care staff was expressed in many places, for example through weekly public displays of applause. This gratitude was based on a recognition that by doing their jobs, healthcare staff were both working in extremely challenging circumstances and also exposing themselves and their families to the risk of COVID‐19 infection.

From the outset of pandemic, researchers around the world sought to describe the impact of COVID‐19 on nurses (Catania et al., 2021; Guttormson et al., 2022; Halcomb et al., 2020; Sugg et al., 2021). As well as identifying pandemic‐specific issues studies reveal the exacerbation and intensification of previously described sources of pressure in nursing: physical, emotional and psychological demands of the job that are incompatible with the resources available (such as inadequate staffing, facilities, and support), creating higher levels of employee stress and risks of burnout (Dall'Ora, Ball, Reinius, & Griffiths, 2020; Greenberg, Docherty, Gnanapragasam, & Wessely, 2020).

The Impact of COVID‐19 on Nursing and Midwifery Workforce (ICON) study was established in March 2020, in the earliest stages of the pandemic and involved the delivery of national surveys at three time points to identify the psychological impact of COVID‐19 on the workforce during and after the first surge of COVID‐19 in the UK. The ICON study revealed that 45% of respondents exhibited symptoms of probable post‐traumatic stress disorder (PTSD), during the first wave of the pandemic. Both personal and workplace factors were associated with adverse psychological effects (Couper et al., 2022). Qualitative interviews with nurses also revealed more explicitly how working during COVID‐19 has impacted on nurses' feelings about their work, their health and emotional well‐being; including moral injury (Ashley et al., 2021; Goh et al., 2021; Greenberg et al., 2020). This research detailed the lived reality behind the statistics with nurses reporting having been forever altered (Maben et al., 2022).

While there is clear evidence of a profound impact of the COVID‐19 pandemic on the nursing and midwifery workforce, less is known about what would have helped nurses at this most challenging of times. Yet, this knowledge is key to ensuring preparedness in the face of future pandemics and other crisis situations. The first of the three ICON surveys (April 2020) had invited respondents to describe the top three things that could be done by the Government or their employer to improve their working life at that time. This paper reports on the prioritized actions that nurses had wanted their employers, or the Government, to take.

## 2 THE STUDY

### 2.1 Aim/s

The aim of this study was to describe nurses' views about how their work lives, during the first wave of the COVID‐19 pandemic, could have been improved. Through a close reading and analysis of their reported views and experiences via free text comments, we set out to better understand factors that influence nurses' work lives and wellbeing, as well as opportunities that exist for improvement.

### 2.2 Design

We undertook a free‐text analysis of qualitative data derived from the first of three national ICON study surveys. The full survey methodology has been described previously. (Couper et al., 2022). In brief, the first ICON survey was an online survey open to responses between the 2 and 14 April 2020. The survey captured information on respondent demographics, their working life and psychological health.

### 2.3 Sample/participants

Study participations in the ICON survey was open to any member of the nursing and midwifery workforce in the UK, both within and outside the NHS. This included registered nurses, health care support workers, students and temporary returnees recruited to expand nursing capacity during the pandemic (Couper et al., 2022). Information about the survey and an internet link were widely distributed through social media (Twitter, Facebook). High‐profile individuals in the UK nursing and midwifery community actively encouraged survey promotion by participants. In addition, survey information was included in emails distributed by key nursing and midwifery organizations (e.g. Royal College of Nursing) and the UK nursing and midwifery regulator (Nursing and Midwifery Council). Access to the survey was open to any individual with the study internet address link. No incentive was offered for completion.

### 2.4 Data collection

The survey was administered through an online survey platform (Qualtrics) and took approximately 15–20 min to complete. Of 3299 survey respondents, 2205 (67%) answered the open‐ended question on improving work lives: ‘Can you suggest the top three things that can be done by Government or your employer to improve your working life right now?’ For the study reported in this paper, we included only those that responded to this question.

The question used was intentionally solution‐focused, asking participants to focus on a limited number of actionable interventions to address factors causing difficulties at work at that specific time. The aims were to go beyond a description of possible problems and negative feelings being experienced (captured in other parts of the questionnaire), to clarify what needed to be done, by inviting a focus on the ‘top 3’ to prioritize the actions *most* needed. The question response was provided in a free‐text format, that allowed respondents to include more or fewer actions.

Additional demographic variables, from other parts of the ICON survey were also included, to allow the respondents' context to better understood and differences explored. These included: gender, age, region, qualification, NHS/other employer and speciality.

### 2.5 Ethical considerations

The ICON study was approved by the University of Warwick Biomedical and Scientific Research Ethics Committee on 27 March 2020 (reference 101/19‐20). Participants gave informed consent through a checkbox at the start of the survey.

This analysis was led by the University of Southampton. Free‐text responses were reviewed for identifiable information; any potentially identifiable information (e.g. place of work) was redacted. The anonymised dataset was securely transferred electronically to the University of Southampton. The current analysis was additionally approved by University of Southampton Ethics and Research Governance Office (Reference: ERGO‐ 61851; approved 07/01/2021).

### 2.6 Data storage

Information was stored in accordance with national legislation and institutional policies. The survey administrator did not limit responses by IP addresses as it was anticipated that participants may complete the survey at their place of work or using a shared home internet router, such that a restriction might prevent completion by eligible colleagues or family members.

### 2.7 Data analysis

A thematic analysis, followed by content analysis (Vaismoradi, Turunen, & Bondas, 2013) of all the responses (thus allowing multiple responses per individual), was undertaken to identify themes, determine frequency of main themes and subthemes, and examine variation between NHS and non‐NHS respondents.

Anonymous free text responses were extracted into a stand‐alone, password‐protected spreadsheet.

A thematic analysis on a sample of responses was undertaken to identify the key themes emerging; these themes were then reviewed to create a classification/taxonomy and produce a coding framework. This was primarily inductive but clearly was also shaped (consciously or subconsciously) by researchers' own experience and knowledge of nurses' working life. This initial thematic analysis became the basis of the coding frame for the content analysis. The coding framework was developed in the following stages:

- first draft was created by the lead investigator (J.E.B.);
- the study researcher (S.A.) tested the framework on a sample of 125 responses;
- a sample of 25 responses were coded by both SA and JEB to test inter‐relater reliability;
- the framework was iteratively refined by updating, re‐testing and updating again, through four cycles, including a second set of inter‐relater reliability testing with KC.

Once satisfied with our working framework the study research team proceeded to code 2055 free text responses. The framework provided a list of main themes and sub‐theme codes which the researchers used to match against the content of respondents' answers to the question. Although the question asked for ‘three things’, every response and issue raised was coded; no limit was applied. Responses could touch on several different themes within one action—every theme alluded to was coded.

Content analysis was undertaken to quantify the frequency that each theme was referred to by respondents. The purpose of the thematic coding and content analysis was to identify common threads in nurses' responses, and patterns of response.

The numerical codes (with the first two digits representing the higher order themes, and the two digits after the decimal places relating to specific sub‐themes within the main theme) in the 10 variables (Code1–Code10) were exported from Microsoft Excel (which was used for coding) to IBM SPSS Statistics for Windows (Version 24.0.: IBM Corp), and using a unique case identifier, linked to a dataset providing background variables for each respondent. The Multiple Response function of SPSS allowed the frequency of each subtheme to be reported, looking across the 10 code variables.

### 2.8 Validity and reliability/rigour

Inter‐relater comparisons and reliability were checked at two stages (Elliott, 2018). During the development phase, a sample of 25 responses was coded by both the lead investigator (J.E.B.) and researcher (S.A.). The purpose of the first round of cross‐checking was to highlight deficiencies and ambiguities in the coding frame. At this first stage, agreement at the main theme level was achieved for 52% responses, full agreement (main and sub‐theme codes) was achieved in 28 out of 62 codes (45%). Following in‐depth discussions and the revisions to the framework to clarify and deal with ambiguity, a code was agreed between JEB and SA on all 62 codes.

A second phase of inter‐rater reliability was undertaken with the finalized coding frame. The codes generated between the researcher and a second coder (K.C.), were compared for responses from 50 participants (which generated 119 codes). Agreement of main theme was achieved on 100 out of 119 codes (84%), and on the subcodes, 65 out of 119 (55%). Following this test, the principal investigator (J.E.B.) acted as arbiter, discussed discrepancies with the team and resolved each discrepancy, to increase the consistency of application of the coding framework to the remaining survey participants responses.

At the end of all coding, the study researcher performed data cleaning by checking codes marked as uncertain throughout the spreadsheet. All remaining coding uncertainties (230 responses out of a total of 5915) were highlighted for the lead investigator (J.E.B.) to arbitrate and resolve any final coding decisions.

## 3 RESULTS/FINDINGS

A description of the profile of respondents is presented in Table 1.

**TABLE 1:** Summary characteristics of respondents^a^

Gender	%	*N*
Female	91.5	1737
Male	8.0	151
Other/prefer not to say	0.5	13
Age (years)		
40 and under	32.7	620
41–50	28.8	546
51–60	31.2	592
61 and over	7.4	140
Area of employment		
Inside the NHS	76.8	1459
Outside the NHS	23.2	440
Professional Grade/Seniority		
Nursing or Midwifery support role	6.5	122
Entry level/staff nurse	27.9	525
Senior staff nurse	27.1	510
Consultant	22.2	417
Highest grade	16.2	305
Speciality		
Primary and Community Care	22.7	431
Acute medicine	9.2	174
Critical care	7.4	141
Research	7.1	134
Outpatients	5.7	108
Acute Surgery	5.6	107
Emergency Department	4.1	78
Elderly Care	3.6	68
Education/HE	3.1	58
Children's Nurse	2.4	46
Operating theatres	2.3	44
Palliative care	2.2	41
Bank/Agency	1.2	23
Midwifery	1.1	20
Learning Disabilities	0.6	11
Other	12.0	228
Redeployment (as a result of COVID‐19, had been or about to be)		
Yes	32.8	623
No	67.2	1274
Infectious disease experience?		
Yes	25.6	487
No	74.4	1412
Overtime worked on most recent shift?		
Yes	57.0	1081
No	43.0	815
Mental health first aid trained?		
Yes	19.4	368
No	80.6	1531

^a^ Respondents who answered the free‐text question at time point 1 survey (April 2020); Missing data are excluded question by question.

The results from the content analysis of responses to the question on actions needed to improve nurses' working lives are presented in Table 2. Our coding frame comprised 101 codes, categorized under 18 main themes. Across the 2205 respondents, we generated a total of 5938 codes (mean average of 2.7 codes per respondent).

**TABLE 2:** Frequency of responses for each subtheme, and grouped by main theme

Over‐arching theme (bold) and subthemes	Number times subtheme cited	% respondents citing subtheme (*n* = 2205)^a^	% respondents main theme^a^
1. Personal Protective Equipment (PPE)/Staff safety			60.0
General point re PPE/Staff safety	456	20.7	
Supply: more, sooner, sufficient, ordering efficiency	515	23.4	
More widely available PPE (e.g. to other key workers)	334	15.1	
Better info. Guidance/consistency re PPE/safety	176	8.0	
Better quality PPE ‐standards; fitting/fit testing	157	7.1	
2. Support to workforce			28.6
General point re support to workforce	161	7.3	
Mental health/wellbeing support	207	9.4	
More training (generally)	119	5.4	
Support for redeployees (e.g. better preparation)	91	4.1	
Better/consistent clinical guidelines	49	2.2	
Individual and team support	37	1.7	
Preparation for critical situation/incidents	36	1.6	
Clinical supervision /reflective practice/circle time—debriefs	22	1.0	
Other specific forms of support, e.g. for returners, students, newly registered and starter, temporary staff, working outside normal speciality, training time/facilities and trauma prevention.	33	1.6	
3. Better communication			21.9
General point regarding better communication needed	101	4.6	
Listen to staff more	49	2.2	
Improve Communication re COVID‐19—Clarity/quality/honesty/timely	316	14.3	
Evidence base for intervention decisions	64	2.9	
Other improved communication—team talks, public communication/guidance, e.g. patients staying at home	6	<1	
4. Pay/Rewards			20.6
General point re improving Pay/Rewards	20	0.9	
Improved/more/better pay	329	14.9	
Paid overtime (currently unpaid)	45	2.0	
Tax relief improvement	42	1.9	
‘Danger pay’; better indemnity cover/Life Insurance	37	1.7	
Reward unspecified or other (e.g. free registration)	37	1.7	
5. COVID‐19 Testing and Isolating			18.3
General point re importance of testing and isolating	147	6.7	
Wider availability/more testing required	182	8.3	
Better testing/antibody testing (e.g. ease of use and usefulness) needed	72	3.3	
Shorter periods of (self) isolation suggested	34	1.5	
Other, e.g. faster testing required, better accuracy of swab testing needed, temperature taking	27	1.3	
6. Staffing and Workload			18.2
General point re staffing and workload as issue	24	1.0	
More staff (general/unspecified/safe levels) required	198	9.0	
Better redeployment; stop inappropriate redeployment; do not remove skills/expertise	111	5.0	
Workforce expansion: enable people to join workforce to help/recruitment	42	1.9	
Reduce admin; less paperwork, fewer emails	30	1.4	
Other, e.g. more speciality‐specific staff (e.g. ICU) or staff with specific skills or levels of experience needed; less pressure/rush; not having to work overtime; better ratios.	48	2.2	
7. Leadership and strategic direction			11.7
General point re Leadership and strategic direction	74	3.4	
Need better Government leadership/policies/lockdown	77	3.5	
Pro‐active leadership (less reactive), e.g. better planning	72	3.3	
Visible leadership/seniors on the floor required	40	1.8	
Other, e.g. empowering staff; less interference from management/more trust	22	1.0	
8. Working hours and rest			10.5
General point regarding working hours and rest	14	0.6	
Ensuring breaks	76	3.4	
More flexibility re work hour	47	2.1	
Rest/time off between shift	31	1.4	
Annual leave (do not cancel/TOIL/extra to thank for C19)	52	2.4	
Rostering/shift patterns/scheduling	43	2.0	
9. Workplace facilities and support for employees			10.3
General point regarding facilities/support	19	0.9	
Access to meals/drinks (especially drinking water)/canteen	53	2.4	
Child/other dependents care	48	2.2	
Showering/changing facilities at work	35	1.6	
Work from home option (if appropriate)	31	1.4	
Other, e.g. transport, staff room/break space (quiet), parking for staff, staff accommodation, access to food/shopping (safely)	84	3.5	
10. Being valued			9.0
General points regarding improvements in being valued	78	3.5	
Recognition	69	3.1	
Not being coerced; less bullying	42	1.9	
Other, e.g. Long‐term respect, being thanked (any method), valuing advanced practice and skills	26		
11. Supplies and resources			8.1
General point re supplies and resources	114	5.2	
Uniforms (supply/laundering)	40	1.8	
Handwashing/sanitizing equipment	31	1.4	
Other, e.g. Drugs (adequate supply) such as Propofol, drugs (pre‐drawn), space/beds, haemo‐filtration equipment (e.g. filter bags)	11	<1	
12. Funding and support for health and social care (beyond acute)			4.3
Adequate funding for health and social care	68	3.1	
Other, e.g. Long‐term funding commitment required, Appropriate resource/use of services (primary care, ED)	32	1.4	
13. Supporting Innovation/new ways of working			2.0
Use of Technology/better IT and sharing lessons learnt	50	2.0	
14. Student issues			1.6
Students—costs of training	16	0.7	
Other student‐related issue	21	1.0	
15. Morale/Team spirit			0.6
General points regarding morale/positive team spirit, encourage/enable good relationships with colleagues/team	13	<1	
16. Other (not covered above, COVID‐19‐related)	97	4.3	4.3
Nothing can be improved	62	2.8	2.8
Total *N* =	5938 citations	2205 cases	>100

^a^ As respondents could give multiple answers, the total percentages exceed 100%.

The results presented in Table 2 present the number of times each issue was cited (column 1), and the percentage of respondents that raised an issue coded to the specific subtheme (column 2). The percentage of respondents citing at least one of the codes in the main theme (looking at higher level that the sub‐codes sit within) are reported in the final column. The results are presented in order by frequency of citation by themes and within themes.

Six themes were referred to by more than 15% of respondents, namely Personal Protective Equipment (PPE)/Staff safety (60.0%), support to workforce (28.6%), better communication (21.9%), pay‐reward (20.6%), COVID‐19 Testing and Isolating (18.3%) and staffing and workload (18.2%). Only 2.8% respondents stated that nothing could be improved. The most commonly cited themes are described in greater detail below, with examples of quotes typical of the theme.

### 3.1 Personal protective equipment and staff safety (ranked #1)

A total of 1638 coded comments related to the need for more or better PPE to keep staff safe—representing the top‐ranked theme. 60% of all respondents cited at least one of the subthemes categorized under PPE/staff safety. This included a need for greater supply, PPE being made more widely available, improving the quality and fit of the available PPE, having better and more consistent guidance on use of PPE, or simply ‘PPE’ – one in five simply said ‘PPE’ without elaborating further. Comments about PPE were commonly related to expressions of fear or anxiety, and a recognition of the mismatch between perceptions of the severity of COVID‐19 and the availability of protective resources:

The obvious is PPE, many staff are worried and anxious. Healthcare workers deaths are being treated like soldiers at war, dying doing what they loved, instead of a failing of the public health system to protect us. [RN (Band 6), NHS Acute Surgery (redeployed); North West England]

This sense of being put in danger at work was repeated by others:

… the government can make as many statements in the news as it wants, we do not have PPE, we don't even have further stocks of hand sanitizer. (…) it currently feels like a lottery (…) ‐ this is our lives. [RN (Band 6), NHS Adult Outpatients redeployed to Acute Medicine; North‐East England]

This translated into a sense of anger at being let down by employers or the Government:

Very evident from rampant healthcare infection rates from COVID‐19 globally that the PPE which PHE [Public Health England] states as guidance, is clearly not sufficient. I would rather honesty, and for them to say sorry we cannot give you the appropriate/enough PPE. And therefore subsequently guarantee that should any harm come to us from COVID‐19 while carrying out our duties there will be death in service pension/benefits to our families. [RN (Band 7), Outside NHS, Community/Primary Care, London, Full‐time Female 26–30]

Finally, there was also a sense of having had to put up a fight to obtain the necessary protection to work safely:

Supply PPE as required to do so without any question or obstacle, each day I have to defend my need for it. People in Sainsbury's [UK supermarket] are better protected than I am! [RN (Band 5), NHS Adult redeployed to Critical Care; South‐East England]

### 3.2 Testing and Isolating (ranked #5)

Closely linked to the issue of protection, or lack of, was a call for increased opportunities to test and isolate, with 462 comments (made by 18% of respondents, ranking #5) related to improvements needed in this area. Nurses called mainly for more testing to be available, and for testing to be made more widely available to all. For example:

Make testing available to me, I have recently had symptoms and my organization would not test me as they said by the time the test returned it would be 7 days. I feel this misses the point….

- Provide testing so that staff know whether they must isolate or not.
- Be clear about testing – there is no stock of COVID‐19 testing swabs for staff
- Stop lying about tests and PPE being delivered and get it delivered.

[RN (Band 8b); NHS Adult medicine (redeployed); North‐East England]

### 3.3 Supplies/resources (ranked #11)

Issues raised within this theme were concerned mainly with supplies and resources (ranking #11); highlighted by 8% of respondents. Again, most comments were related to the COVID‐19 context specifically, for example, the need for uniform laundering, greater availability of uniforms, sanitizing equipment, filters and filter bags [haemo‐filtration equipment], pre‐drawn drugs. Some supply issues were more generic, such as the need for more beds, more clinical working space or more readily available supplies and resources generally.

…However, we have been told we are not allowed to wear scrubs due to a shortage. So, in certain situations such as staff having to leave the ward to go down to blood bank staff have no change of clothing and therefore infection control policies has been breached [RN (band 6), NHS Adult Acute surgery, East Midlands]

This issue also related to infection prevention concerns:

Give us all disposable scrubs as we have to wear our uniforms (…) then take them home and wash them (my family are at high risk). [RN (band 6), NHS adult Emergency Department, Scotland]

As well as statements about not having enough basic equipment to carry out clinical nursing care safely and effectively:

Enough equipment ‐ ventilators, infusion pumps, monitors, drugs…. [RN (band 7), NHS Adult Critical Care, South East England]

A notable strength of feeling about the risk to nurses' health, and even survival, was also evident:

‘….Recognise the nurses (are) dying and putting themselves on the line, and the media can perhaps speak to people and recognise that it isn't just ventilators but a whole load of other equipment that potentially could be needed’ [RN (band 6), NHS Adult Outpatients, South East England]

### 3.4 Support for staff (ranked #2)

The second mostly frequently cited theme (highlighted in 674 responses and cited by 28.6% of respondents) related to better support for staff in general, or for particular groups within the nursing workforce. The most frequently cited specific action was better support for mental health and wellbeing (cited 207 times, by 9.4% of respondents). Most respondents called for attention to be paid to specific groups—most notably staff who had been redeployed (working outside usual role/area/patient group) to a different clinical area, as a result of managing influx of COVID‐19 patients (91 citations, by 4.1% respondents). Whilst specific training and support was called for, 119 comments related to more training be available generally.

*Quote examples*:

re the mental health scheme. It's all well and good everyone trying to boost our morale and support us, but at the end of the day, the shifts are gruelling, back breaking and emotionally exhausting. Our mental health is suffering, and a link to the wellbeing nurse isn't good enough…. [RN (band 5), NHS Adult medicine, South West, Full‐time, Female 26–30]

Improved coordination of redeployment. I'm more than happy to help and had offered to be redeployed, but in one day six different senior nurses contacted me, by phone and email to organise my moving to ITU. I found this stressful… [RN (band 7); NHS Adult medicine, East of England, Part‐time, Female 36–40]

Better support and training for new starters, preceptees, and specialty nursing. Better training for agency and bank staff ‐ that they do not have to pay for themselves and suits the specialty if they work in it regularly! Sorry… four things ‐ better mental health support! [RN (Band 5); NHS Adult medicine, Bank/agency contract, Female 31–35]

### 3.5 Better communication (ranked #3)

As well as the need for information to support redeployment there was a general sense of ‘information overload’ being a problem. Staff highlighted the need for information to be channelled through fewer sources, provided verbally where possible and in a timelier way ‐ although it was recognized that delays were often related to national issues or shifting Government regulations, rather than local delays or changes in practice or guidelines.

This was exemplified by comments from several participants:

Cut down on all the communication sources, as so many e‐mails can cause confusion ‐ also take time to read as so busy. Can advise be summarised by Infection Control direct to front line staff [RN (band 5), NHS Community/primary, East Midlands]

Channels commonly being used in the UK NHS were also criticized:

To update staff at home. Currently all information is via internal emails/intranet which cannot be accessed at home. Therefore communication is poor, disjointed and delayed [RN (band 5), NHS Acute Medicine, West Midlands]

### 3.6 Pay and Reward (ranked #4)

Comments in this theme reflected a general dissatisfaction with the level of pay for working in such a high‐risk occupation during a pandemic; with higher workloads, often increased responsibility and a high level of emotional burden. Respondents called for financial reward and recognition for the significant contribution they made to the pandemic response, particularly those who had felt less well appreciated during this time (e.g. those working in care homes and other community settings).

Comments focused on pay specifically:

Improve pay in recognition of the skills, demanding emotional work and high level of risk we carry accountability for [RN (band 5), Mental Health, South West]

Recognise NHS staff with substantial pay reward for example lump sum payment 1000 as other people are rewarded 80 per cent of their pay for doing no work and we are working our normal hours plus 75 per cent extra hours with no reward [RN (band 7), ‘other’ Adult nurse setting, South East]

As well as highlighting areas where less attention had been paid:

Provide extra pay for those who are still working with less staff less resources, but same or higher level of residents' needs. It's become normal for a nurse to be a carer, cook and maintenance man at the same time. Without any thanks or any clapping from the public on Thursday – care homes matter as well [RN (band 6), Care/nursing home, Scotland]

### 3.7 Staffing and Workload (ranked #6)

Respondents also raised concerns about approaches to staffing and organization of care during the first surge of COVID‐19. They reflected on the need to capitalize on the existing as well as the next generation nursing workforce as priority. This is to maximize the retention of experienced nurses and to prioritize training of the future health and care workforce. The impact of challenges on workload and staff wellbeing was evident in these responses.

One respondent made this in a very clear way:

Increase number of staff on shift (so that we can all take breaks and go home on time) [RN (band 5), Mental Health, South West]

There was also cynicism about the newly constructed Nightingale hospitals that could not be staffed, without having to move people from already heavy workloads:

Train up 43,000 nurses to fully staff the NHS before they think they can magic up new nurses for all the nightingale wards [RN (band 5), Critical care, North East]

The sense of frustration with how decisions were taken also presented in these comments:

I've seen senior managers undertake clinical training irrelevant to them, whilst clinical staff who have suspended their routine work await re‐organization. This approach has frustrated staff. Can the managers please manage! [RN (band 7), Research, East Midlands]

### 3.8 Overview: Tone of responses

Despite the focus on priorities for action, the strength of feelings associated with multiple workplace problems was evident in the way in which many respondents answered these questions – both in terms of what was expressed (exasperation, outrage, fear, anger, disappointment) and in the manner in which views were expressed, for example use of capital letters, repeated points and exclamation marks, for example: ‘. PPE, PPE, PPE!!!!!!!!’. It seemed that many respondents used this opportunity to vent their emotional response about the actions that felt were needed urgently.

### 3.9 Differences by employer: NHS Vs non‐NHS

Findings in Table 3 show the proportion of respondents that cited an issue related to each of the main themes, between those respondents working in the NHS, compared with those working elsewhere. Workforce support, including consideration of working hours, facilities and pay/rewards were cited by a larger proportion of nurses in the NHS, and were seen as requiring improvement. A larger proportion of those working outside the NHS referred to testing and isolating, as well as PPE & staff safety. PPE and staff safety were the most frequently cited issues requiring action overall, regardless of employment setting and was cited by 60.2% of respondents within the NHS and 63.4% in non‐NHS settings. Overall respondents in both NHS and non‐NHS employment had more in common than differences; it was only when comparing the frequency of comments raised that related to workforce support, working hours, workplace facilities and student issues did show statistically significant differences (using chi‐square test).

**TABLE 3:** Percentage of respondents citing each theme by sector where working

Theme	Number citations	% cases (all)	Rank order	NHS (%)	Outside NHS (%)
PPE and Staff safety	1423	60.0	1	60.2	63.4
Workforce support^a^	674	28.6	2	30.7	25.9
Better communication	470	21.9	3	22.1	23.0
Pay/rewards	450	20.6	4	21.7	19.1
Testing and isolating	415	18.3	5	18.7	20.9
Staffing and workload	381	18.2	6	18.3	16.4
Leadership and direction	251	11.7	7	12.0	12.0
Working hours^a^	229	10.5	8	11.7	7.7
Workplace facilities^a^	247	10.3	9	11.6	8.2
Being valued	191	9.0	10	9.1	10.0
Supplies and resources	169	8.1	11	8.6	6.6
Support for health and social care	86	4.3	12	3.9	5.7
Other	74	4.1	13	3.8	4.1
Nothing can be improved	52	2.8	14	2.5	3.4
Supporting innovations	43	2.0	16	2.0	3.0
Student issues^a^	33	1.6	17	1.2	3.0
Morale/team spirit	12	0.6	18	0.7	0.5
Leaving/retiring	4	0.2	19	0.1	0.5
Total (*N* = citations)	5204				
Total (*N* = cases/respondents)	1899			440	1459

^a^ Differences between NHS and non‐NHS significant in chi‐square (*p* < .05).

## 4 DISCUSSION

Survey respondents are often asked to give their views through open‐ended questions with free‐text boxes, but rarely are these types of data utilized fully (Decorte et al., 2019). Through this analysis of their own words, we sought to better understand the nature of the working‐life priorities for nurses during COVID‐19 (during the peak of the first wave in the UK) and to enable the nurses' perspectives to be heard and utilized to inform future pandemic responses.

In this study, we reported on nurses' suggestions for priority actions required of the Government and employers to immediately improve the working lives of the UK nursing and midwifery workforce during the early stages of the COVID‐19 pandemic. Of the 2205 respondents received, over 97% identified at least one priority action. By far, the most common action is linked to the provision of PPE. We identified some small differences in priorities between those working in the UK NHS and those employed in the non‐NHS UK health sector.

The question focussed specifically on actions required ‘right now’ to improve the working lives of respondents. During April 2020, during the first wave of the UK COVID‐19 pandemic, respondents' primary focus was on personal protective equipment, reflecting both the emergency context of healthcare delivery during the early stages of this pandemic and problems with provision. A core concern in responding to the pandemic was ensuring adequate supplies of PPE so that health care staff could continue to work safely. In March 2020, ‘in response to UNISON's concerns, NHS England and NHS Improvement announced that there has been an increase in PPE being delivered for use by frontline healthcare workers in England’ (UNISON, 2020). The UK Government reported on attempts to secure procurement of (PPE) at the outbreak of the pandemic, and stated in November 2021: ‘we continue to stand by the efforts we made at the height of the early pandemic to prioritise and protect our staff in the frontline’ (Gov.UK, 2021).

Despite these assurances, there were widespread reports of individual nurses and midwives being asked to ration PPE use, or even to make their own PPE (e.g. using bin‐bags in place of gowns, a finding particularly common in community and mental health settings). At the same time, there were also increasing reports of healthcare workers, including frontline nurses, dying from COVID‐19 infection. For those in Government, there was a need to source PPE supplies urgently and to ensure that (sometimes scarce) available resources were used effectively. A key challenge was the pervasive uncertainty about the precise nature of risk faced during different clinical activities, and the variations in clinical guidelines that were being produced.

The scarcity of PPE for some nurses and midwives, and requests to ration its use, aligns with the findings from other UK studies and international studies (Catania et al., 2021) (Hoernke et al., 2021). Research based on testimonies from 23 nurses in Italy during April and May 2020, highlighted the huge impact of COVID‐19 on the Italian nursing workforce, and revealed the high risks associated with caring for COVID‐19 patients; risks which had increased as a result of shortages of appropriate PPE (Catania et al., 2021). In the UK, an early assessment in March 2020 reported inappropriate provision of PPE, as well as inadequate training and inconsistent guidance that served to hamper health care workers further (Hoernke et al., 2021). Despite widespread criticism of UK employers and the Government's response in relation to PPE supplies, there was also some evidence of rapid improvement. For example, between April and May 2020, when the first two ICON surveys were disseminated, the proportion of respondents agreeing or strongly agreeing that correct PPE was always available increased from 39% to 60% (Couper et al., 2022).

According to this research, the evident neglect of a fundamental aspect of employee safety, in the form of available PPE for nurses in the UK in April 2020, was compounded by other basic being unmet and requiring action. They included: sufficient staffing and time to be able to take breaks during a shift to eat, drink and be refreshed; feeling valued and respected; good people management with clear and transparent communication; flexibility and choice in working hours and being able to take annual leave; being paid well for the work done (including overtime); having access to basic workplace facilities such as parking, somewhere to change at work, a canteen or access to hot meals during long shifts. Issues which have been recorded by large‐scale surveys of nurses that pre‐date the pandemic (McIlroy, 2019).

Enshrined in health and safety legislation, safety at work is a legislative right for all employees (Gov.UK, 2019). In the UK, the NHS ‘Workforce Implementation Plan’ (NHS, 2019—Unspecified) and more recently the NHS England ‘People promise’ developed as part of the ‘Looking After our People’ workforce strategy pledges to ‘provide better mental health and wellbeing support to NHS staff’. This includes the provision of appropriate clothing and personal protective equipment.

Maslow's seminal ‘Hierarchy of Needs’ sets the foundation for understanding that we all have fundamental needs that must be met to achieve our full potential, and that there is a hierarchical sequence towards understanding and meeting these (Maslow, 1943). Achieving the top tier of ‘self‐actualisation’ depends on firstly meeting basic physiological needs (for shelter, food, water and clothing) and being safe. In this theory, meeting these essential needs is necessary before higher order needs, for love and a sense of belonging; then esteem, and finally being able to achieve one's full‐potential, can be addressed. It is striking how many of the responses from nurses in our analysis related to these most basic of needs not being met: to feel safe, or to satisfy the physiological need for food and water.

Kahn's (1990) grounded theory approach to understanding the differences between engaged and disengaged workers helps to recognize further the importance of individuals' psychological safety needs. The concept has been developed further by Edmondson who suggests that ‘psychological safety’ is the need for individuals to feel safe to speak up with ideas, questions, concerns or to report mistakes, without fear of punishment or humiliation (Edmondson, 1999). She discovered that those teams with better outcomes were those most likely to admit more mistakes, whilst the teams with fewer good outcomes were more likely to hide errors. Edmondson postulated that psychological safety was a key factor in team performance. This was never more important than during the critical phase of the COVID‐19 pandemic—yet difficult to achieve (Adams et al., 2020).

Psychological safety is complex in the workplace and depends on the existence of trust between workers and employers. Staff feeling that they are being put at risk and are insufficiently protected, both physically and psychologically, can negatively impact the relationships and trust between nursing staff and their employers and may lead to possible longer‐term damage in terms of staff motivation, morale and retention. This is likely also to affect the ability of nurses to provide empathic and caring support for patients. Recent work has also highlighted how nurses and midwives did not always feel safe to speak out about shortages of equipment or of staff, with some being told to stop speaking to the media (Conolly et al., 2022). Others have argued that the nursing voice nationally and internationally was ‘on mute’ and that organizations and governments were deaf to nurses' experiences and to the issues raised (Rasmussen et al., 2022).

Lack of adequate PPE has far‐reaching impacts on nurses and all frontline workers—exposure to disease and death is the more immediate consequence, but further potential impacts such as mental health, psychological safety, broken bonds of trust, reducing morale and eventually further staff shortages can pose long‐term damage to health workforce and thereby, the safety of health services delivered to patients. The failure to provide adequate personal protective equipment to health and social care workers during the pandemic has been described as being indicative of ‘the disintegration of any culture of integrity, transparency, honesty, and support for healthcare staff from the government and NHS employers’ (Oliver, 2021). It has also prompted nurses to question why they are perceived as expendable, or why the image of nursing prevents their concerns being taken seriously (Bennett, James, & Kelly, 2020).

Chan makes this assessment of the PPE shortages: ‘While the negligence may be arguably excused during crises, the failure to meet the basic resourcing needs of frontline healthcare workers has breached the minimum standard and ethical imperatives in protecting them from life‐threatening harm while they continue to treat an increased influx of patients’. And goes on to conclude:

‘The state may not be able to salvage the deaths and distress caused to frontline healthcare workers, but it can act more substantively to protect them and to restore public trust that the healthcare system would not collapse in times of pandemic. It has been argued here that hospitals ought to maintain their obligations to provide PPE to healthcare workers, because a failure to adequately protect them is also a failure to protect public health’

p. 202 (Chan, 2021).

It is not just the availability of PPE that impacted on nurses' health and psychological safety—pay, overtime, lack of training (use of students) and organizational changes (redeployment) during the pandemic were sources of strain that require action, according to these participants. Workplace performance is strongly correlated with employee trust (Brown, Grey, McHardy, & Taylor, 2015); unpaid overtime and limited access to training are likely to erode employee trust, and reorganization, experienced at either the employee (e.g. redeployment) or organization level (e.g. service reconfigurations), can increase stress (Pollard, 2001).

How the unmet need for workplace protection and safety for nurses was handled is likely to have caused lasting damage to levels of trust and goodwill that may already have been strained. During the inquiry into procurement at the start of the Covid: The Secretary of State for Health, told fellow members of parliament that despite ‘local problems’ there was ‘never a national shortage of PPE’ (Nursing‐Notes, 2021). This account of PPE shortages differs hugely from the lived experience of nurses, both in and outside of the NHS, 60% of whom said more or better PPE was needed. While many studies about the lack of PPE during COVID‐19 have focussed on the risk of infection this study demonstrates that deeper psychological factors can be impacted in terms of trust in political institutions. Undermining of trust and developing a sense of betrayal could have long‐term detrimental effects on employer/employee and institutional betrayal, which is difficult to row back from and repair (Gold, 2020). The pandemic, and responses to it by employers and government, has shaken a potent cocktail of discontents and has, for many, breached or broken the psychological contract between an employee and their employer—that is the unwritten, intangible agreement that that makes up their relationship. Such violation can lead to staff feeling angry and betrayed (Morrison & Robinson, 1997). The example of PPE is only one of many that were symbolic of nurses feeling abandoned to their fate, rather than feeling that the Government had their safety in their sights.

In the UK, there have also been long‐standing concerns linked to staffing, workforce support and pay. These have been linked to ongoing high rates of workforce attrition, which have accelerated over the course of the pandemic (Cambell, 2022). These issues were also cited frequently in the ICON survey responses but received less focus than in other frequently cited workforce surveys—perhaps due to the fact that this question asked about immediate improvements that could be made, not work life concerns in general.

### 4.1 Limitations

Important over‐arching limitations, such as the small self‐selecting sample that may not be representative of the entire UK nursing and midwifery workforce, are described in the initial ICON survey study publication (Couper et al., 2022). This analysis has several additional limitations. First, it was not practical, due to the amount of data, for each response to be coded independently by two researchers. However, for the sub‐sample that was coded by two individuals, we achieved substantial agreement, particularly for the main code. Second, we determined that the most appropriate way was to allocate as many codes as appropriate to each action identified within the response. This meant that where a response referred to three similar actions (e.g. PPE, PPE and PPE) it will have been coded multiple times with the same code. This may, in part, explain the focus on PPE as the most frequently cited code. Third, our sample included only the 66% of individuals accessing the survey who responded to this specific survey question. Some non‐respondents may not have reached this question within the survey. For those that did, but chose not to respond, it is not clear whether their non‐response reflects the view that there were no actions to be taken that might have improved their working lives.

## 5 CONCLUSION

As we look forward and hope for the imminent arrival of a post‐pandemic period of recovery, these findings should be seen as having ongoing relevance to nursing and health services more generally. We may risk dismissing these findings as being only of ‘historical interest’, at our peril. Placed in the wider context of the ICON survey findings, and of the national and international nursing labour market, this is not simply about particular needs being expressed by nurses that were not met at a particular time. Failure to ensure that nurses, the group of staff most relied on to provide care and valued above all others (IPSOS, 2021), are working safely and that they feel safe. This represents a serious breach of the psychological contract, and thus a violation of the trust, between nurses and not just their employers, but also in terms of the fabric of a national health service and Government policies, as well as the resourcing decisions on which health and social care provision depends.

This article opened with a reflection on the now recognized need to expand and develop the nursing workforce; to sustain and retain nurses globally and nationally (Buchan et al., 2022). In England, the Government have pledged to increase registered nurse numbers by 50,000 between 2020 and 2024. The faith of many nurses has been shaken, as the respondents in this study have shown. Their fundamental needs to work safely were not always met during the COVID‐19 pandemic. We know already that nurses and midwives have experienced long‐term mental and physical health consequences and the numbers of leavers have increased (Cambell, 2022). Waiting to see what the full repercussions of the unmet needs of the nursing workforce, as well as the outcomes of COVID‐19 more generally, might be a high‐risk strategy. This is true, both in terms of the current and future nursing workforce, but also in societal terms, as we seek to staff health and social care services with motivated nurses for the years ahead.

## AUTHOR CONTRIBUTIONS

All authors have made a substantial contribution to the concept or design of the article, or the acquisition analysis or interpretation of data for the article, drafted the article or revised it critically for important intellectual content, approved the version to be published, agreed to be accountable for all aspects of the work in ensuring that questions related to the accuracy or integrity of any part of the work are appropriately investigated and resolved.

## FUNDING STATEMENT

The full ICON study was supported with funding from the Burdett Trust for Nursing.

## CONFLICT OF INTEREST

All authors confirm no conflict of interest.

### PEER REVIEW

The peer review history for this article is available at https://publons.com/publon/10.1111/jan.15442.

## RETENTION OF RIGHTS

For the purpose of open access, the author has applied a Creative Commons attribution licence (CC BY) to any Author Accepted Manuscript version arising from this submission.

## Data Availability

The datasets generated during and/or analysed during the current study may be made available by the corresponding author on reasonable request.
